# A Case Report of Brown Recluse Spider Bite

**DOI:** 10.7759/cureus.16663

**Published:** 2021-07-27

**Authors:** Basel Abdelazeem, Kianna Eurick-Bering, Sarah Ayad, Bilal Malik, Zirka Kalynych

**Affiliations:** 1 Internal Medicine, McLaren Health Care, Flint/Michigan State University, Flint, USA; 2 Internal Medicine, Michigan State University College of Human Medicine, Flint, USA; 3 Internal Medicine, Rutgers New Jersey Medical School/Trinitas Regional Medical Center, Elizabeth, USA

**Keywords:** brown recluse spider, spider bite, cellulitis, necrotic ulcer, case report

## Abstract

Brown recluse spider is a spider of the genus *Loxosceles and* also known as violin spider or fiddle-back spider. Brown recluse spider is characterized by having six eyes, with a pair in front, a pair on both sides, and a gap between the pairs. The other spiders have eight eyes in two rows of four. Brown recluse spider bites are challenging to verify but may be clinically diagnosed with consideration of geographic location, seasonality, and clinical characteristics. We present a case that involves a brown recluse spider bite in a 59-year-old female with malnutrition and polysubstance use who developed systemic symptoms and a dermonecrotic wound. Local wound care and intravenous (IV) antibiotics lead to clinical improvement by hospital day three, at which time the patient left against medical advice. The case highlights the challenges of diagnosing a brown recluse spider bites, particularly in a patient with multiple risk factors for necrotizing soft tissue infection. Furthermore, the present case represents one of the few case reports of a brown recluse spider bite in Michigan.

## Introduction

Brown recluse spider can be found worldwide and in the United States mainly in the south from Texas to Florida. Due to recent climate change, the brown recluse spider started appearing in other states. Brown recluse spider is usually hiding in dark locations such as under boxes, piles of rocks or leaves, and other dry, dark, and warm locations [1, 2]. Spider bites in humans can cause dermonecrosis, hematological abnormalities, and renal failure. Diagnosis of brown recluse spider bites is challenging and usually presumptive through the clinical picture and epidemiological information as only a few patients bring the spider with them for identification [2]. Our patient was a 59-year-old female who developed systemic symptoms, and a dermonecrotic wound likely secondary to a brown recluse spider. She reported that an insect bit her left leg while she was sleeping in a park. Our patient is one of the few cases reported in Michigan. Our case reviews the different presentations of a brown recluse spider bite and raises the awareness between the physicians regarding including spider bite in the differential diagnosis of dermonecrosis.

## Case presentation

A 59-year-old female with a past medical history of an alcohol use disorder, polysubstance abuse, and malnutrition (BMI: 17.6) presented to a Michigan hospital in June with a five-day history of a painful left lower extremity wound. The patient reported an insect bite to her left lower extremity while sleeping in a park with the development of immediate pruritus, redness, and mild swelling at the bite site. There was no history of trauma to the area.

The next day, the patient developed a blister associated with pain and redness at the bite site. The pain was stabbing and of 9-10/10 in severity. Two days before the presentation, the blister ruptured and developed into a black eschar. The patient reported associated fever, diaphoresis, nausea, and multiple episodes of diarrhea daily for 3-4 days. Social history was pertinent for smoking one pack per day for 20 years, occasional cocaine usage, and heavy alcohol use, drinking a fifth of vodka daily for 11 years. The patient denied any history of IV drug use or skin popping. The patient denied any travel history, and she lives in Michigan only. The patient denied any other past medical history including diabetes mellitus, hypertension, cardiovascular disease, and chronic obstructive pulmonary disease (COPD). The patient was not on any antiplatelet or antithrombin therapy.

**Figure 1:**
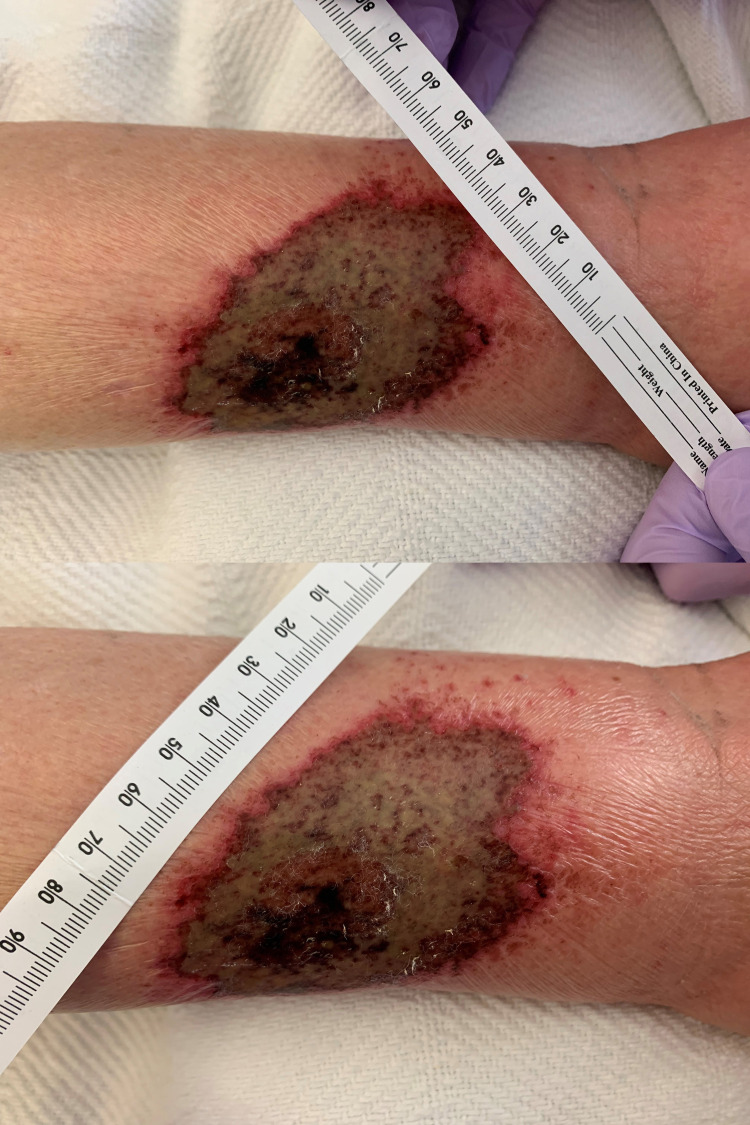
Necrotic skin wound. Necrotic skin wound on the lateral aspect of the left lower third of the leg, measuring 5 x 10 centimeters, associated with swelling, erythema, and warmth extended to the dorsum of the foot.

**Figure 2:**
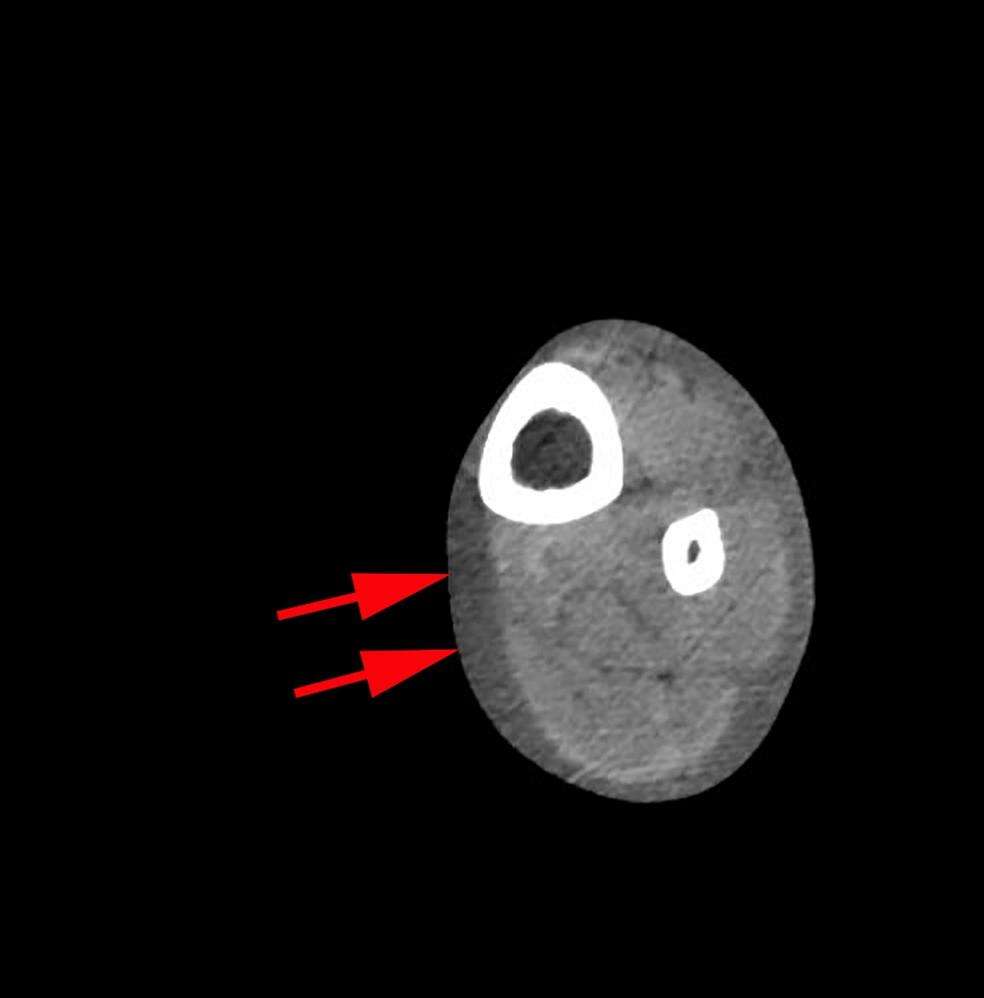
CT scan of the left lower extremity. CT of the left lower extremity revealed diffuse subcutaneous edema with fat stranding (red arrows), suggestive of cellulitis, but was negative for necrotizing fasciitis or osteomyelitis.

On examination, the patient had a necrotic wound on the lateral aspect of the left lower third of the leg, measuring 5 x 10 centimeters, associated with swelling, erythema, and warmth extended to the dorsum of the foot (Figure 1). There was significant tenderness associated with palpation. Laboratory workup is summarized in Table 1. The urine drug screen was positive for cocaine, opiates, benzodiazepines, and cannabinoids. Initial differential included cellulitis, necrotizing fasciitis, and a brown recluse spider bite. Due to concern for staph infection or necrotizing infection and the patient’s history of substance use, the patient was started on broad-spectrum antibiotics including IV Aztreonam 1 gm Q8 hours, vancomycin 1000 milligrams (mg) once, and clindamycin 600 mg Q8 hours. CT scan of the leg was ordered to rule out necrotizing fasciitis. CT demonstrated diffuse subcutaneous edema with fat stranding, suggestive of cellulitis, but was negative for necrotizing fasciitis or osteomyelitis (Figure 2). A methicillin-resistant Staphylococcus aureus (MRSA) screen and the blood culture were negative. Vancomycin and aztreonam were discontinued, only IV clindamycin was continued. General surgery was consulted for possible wound debridement and recommended local wound care as well as continued antibiotics.

**Table 1:** Laboratory workup. H: High; L: Low; BUN: Blood urea nitrogen.

Lab	Value	Reference range
WBC count	12.62 H	4.50-11.00 X 10*3/uL
Hemoglobin	12.2	12.0-15.7 g/dL
Platelet count	176	140-440 X 10*3/uL
ABS neutrophils	8.82 H	1.40-6.50 X 10*3/uL
Creatinine	0.91	0.50-1.50 mg/dL
BUN	16.0	9-27 mg/dL
Albumin	2.3 L	3.8-4.9 d/dl
C-reactive protein	1.9 H	0.0-0.9 mg/dL

On hospital day 3, the patient was clinically improved with receding erythema localized to the wound edges with viable tissue and a small necrotic wound center. The patient was still complaining of severe pain at the site of the bite, but nausea, diarrhea, fevers, and chills had resolved. The patient left against medical advice on hospital day 3. Before leaving, she was counseled on the risks of leaving against medical advice and encouraged to return to the hospital if she develops systemic symptoms of infection or worsening of her wound.

## Discussion

The brown recluse spider is found in the southern and central regions of the United States [3]. Reports of brown recluse spider bites demonstrate seasonality, with 96% of cases in the United States occurring between April and October [3]. Therefore, clinical characteristics, seasonality, and geography can provide clues to the diagnosis of suspected brown recluse bites. However, confirmation of a brown recluse spider bite requires physical evidence of the spider [4]. Clinically, the bite of a brown recluse begins with a small, central blister surrounded by erythema and purpura [4, 5]. Within 48-72 hours, a necrotic ulcer with central eschar may develop [4, 5]. The necrotic nature of the wound is mediated by an immune response to phospholipase D toxin, which is found in the spider’s venom [6].

Brown recluse spider bites have four distinct clinical presentations [7]. First, the bite can be unremarkable, with minor local damage and a lesion that heals spontaneously. Second, a mild presentation has been described with a small lesion with surrounding erythema and pruritus. The third presentation is skin necrosis. Lastly, the most severe presentation includes systemic involvement with vascular system effects, including disseminated intravascular coagulation (DIC) [7]. Although there is no standardized treatment, the typical treatment of brown recluse bites includes local wound care and systemic antibiotics [4]. More severe cases may require skin grafting and the use of hyperbaric oxygen therapy [4].

The present case demonstrates the challenges associated with the diagnosis of brown recluse spider bites. The patient had many risk factors for necrotizing soft tissue infection, including her age, malnutrition, and substance use. Initially, a brown recluse spider bite was not suspected until the patient reported a bite by an insect at the site of the necrotic wound that began with pruritus and erythema and then developed into an eschar. In cases where a patient has significant risk factors for necrotizing soft tissue infection, a careful history is required to determine if the patient reports a bite that may suggest a brown recluse spider bite when in the correct seasonality and geographic range. 

While brown recluse spiders have traditionally been found in the southern and central US, the presence of brown recluse spiders in Michigan has been attributed to climate change. Due to global warming, the climate in Michigan has warmed 2-3 degrees (F) in the last century. As a result, cases of brown recluse spider bites in Michigan are starting to rise. In addition, although rare, the patient demonstrated symptoms of systemic involvement, including subjective fevers and chills, nausea, and diarrhea. Usually, systemic complications such as hemolytic anemia, DIC, and acute renal failure may not appear until around day five after brown recluse bites [1, 4]. As a result, patients should be counseled on the potential to develop complications even a week after a brown recluse bite, and regular follow-up should be scheduled for re-assessment and management.

## Conclusions

Due to climate changes, the number of brown recluse spiders is increasing in Michigan. Patients with significant risk factors for necrotizing soft tissue infection complicate the diagnosis of a suspected brown recluse spider bite. A careful history is crucial to determine if the patient reports a bite. We presented a case of a 59-year-old female with malnutrition and polysubstance use who developed systemic symptoms and a dermonecrotic wound secondary to a brown recluse spider bite. The present case represents one of the few case reports of brown recluse spider bites in Michigan and highlights the challenges of diagnosing and different clinical presentations of brown recluse spider bites.
